# Leaving professional competition on the field: Professional collaboration in promoting college athlete mental health

**DOI:** 10.3389/fpsyt.2022.1079057

**Published:** 2022-12-08

**Authors:** Matt Moore, Paul Gorczynski, Cindy Aron, Payton Bennett

**Affiliations:** ^1^Department of Social Work, Ball State University, Muncie, IN, United States; ^2^School of Human Sciences, University of Greenwich, London, United Kingdom; ^3^Private Practitioner, Chicago, IL, United States

**Keywords:** student-athlete, mental health, interprofessional practice, integrated care, sport

## Abstract

The wide range of challenges facing college athletes often results in the need for micro and macro mental health services. This article examines a competency-based model of integrated care. A team of mental health professionals must be intentionally created to support athletes throughout various aspects of their unique experience. Interprofessional practice benefits college athletes by providing them with a broad spectrum of care throughout their college experience.

## Introduction

Recent studies show college athletes are susceptible to mental health symptoms and disorders such as depression, anxiety, mood disorders, substance abuse and use, and eating disorders (1–4). Complicating our understanding of college athlete mental health are a variety of individual and environmental factors. For example, some college athletes are under the spotlight for sexual assault and interpersonal violence (5), sport specific stressors [e.g., injuries, multiple surgeries, decreased performance, relocation, being away from home for long periods, and maladaptive perfectionism; (3)], criminal justice involvement (6), adverse childhood experiences (7), learning disabilities and other academic concerns (8), racial and sexual injustice (9, 10), and other health and safety worries. There is also an increased vulnerability, yet resiliency, which means college athletes can be mentally unwell and still engaging in successful psychological strategies in their sport. This is compounded by the reluctance many athletes have to seeking help [e.g., fear of being perceived as weak and repercussions of disclosure; (11, 12)].

With a rise in discussion regarding college athlete mental health, comes an increased need for professionals who can provide micro and macro-based services to college athletes and those entrusted with their care. This care requires an integrative approach that encourages interprofessional practice and education (1) and draws upon the strengths of athletic trainers, sport administrators, sport psychiatrists, sport psychologists, sport social workers, and other licensed mental health professionals. The development of an interprofessional model promotes social justice and social change by focusing on the unique needs of athletes at both an individual and an environmental level (13). An interprofessional care team must (1) believe in helping college athletes to address the mental health needs impacting their abilities to be successful both in and away from competition, (2) ensure access to education and services to address mental health symptoms and other identified stressors, (3) remain mindful of the individual and cultural factors each college athlete brings to competition and their life aspirations, and (4) understand the impact both athletic and non-athletic supports have on a college athlete's mental health and wellbeing (1, 13–15). To promote these outcomes, members of an interprofessional care team must be competent in various constructs and perspectives that support the engagement, teaming, assessment, planning, and intervention of college athletes when they are experiencing mental health symptoms and disorders.

According to the Interprofessional Education Collaborative (16), there are four core competencies for interprofessional collaborative practice. Members of an athletic interprofessional care team must embrace these competencies to best promote the mental health of those involved in college sports. These competencies include: (1) working with individuals of other professions to maintain a climate of mutual respect and shared values, (2) using knowledge of one's own role and those of other professions to appropriately assess and address mental health care needs, (3) communicate with college athletes, the sporting community, and professionals in health and other fields in a responsive and responsible manner that supports a team approach to the promotion and maintenance of behavioral health, and (4) apply relationship-building values and the principles of team dynamics to perform effectively in different team roles to plan, deliver, and evaluate programs and policies impacting college athlete mental health (16).

While many college programs across the globe embrace an interprofessional model of care, there are still barriers that can minimize effectiveness. The greatest of these barriers is interprofessional competition (17). We do not have to search far to find a world where turf wars exist within various professions engaging in the space of college athletics (18, 19). One could argue these professions are protecting lucrative functions of their field. Others may say such competition is contrary to the ethical codes of helping professions, creating a commodification of the athlete that detracts from the larger goal of providing extraordinary care to vulnerable college athletes. Regardless of reasoning, athletes need support staff that work together as opposed to making life more complex. For these reasons, college athletic programs should embrace a competency-based model for providing interprofessional care to their athletes.

## Competency-based interprofessional collaboration for college athletes

The first competency explores the values and ethics of each member of the interprofessional care team (16). Members of the interprofessional care team should have a common respect for each other and for privacy and confidentiality in the service delivery process. Furthermore, there should be a respect for the cultures, values, roles, and expertise of all professions working to improve college athlete outcomes. If all members of the interprofessional care team maintain competence in their own practice area and act with integrity toward others, it helps minimize ethical dilemmas and creates clear trajectories for each member of the team. Informational diversity can in turn, maximize the collective intelligence of the care team.

The second step in supporting a competency-based approach is clearly identifying the roles and responsibilities of the members of an interprofessional care team (16). This approach encourages open conversation about skills, knowledge, abilities, and limitations. Professionals must also engage in conversations about how best they can complement each other and their professional expertise. Embracing such an approach can promote the safe, timely, efficient, effective, and equitable approach of college athlete care. Furthermore, having clearly defined roles and responsibilities allows for clearer communication and supports engagement with diverse professionals within and outside of the athletic setting.

The third competency builds on the aforementioned interprofessional communication (16). Members of an athlete's interprofessional care team must trust each other when delivering knowledge and opinions to team members involved in athlete care. The knowledge of each team member provides a holistic approach for understanding information, treatment, and care decisions to promote college athlete mental health. By learning from one another, members of the interprofessional care team encourage diversity of thought that recognizes the uniqueness of each profession, promotes crucial conversation, and maintains an overarching emphasis on athlete-centered care.

**Figure 1:**
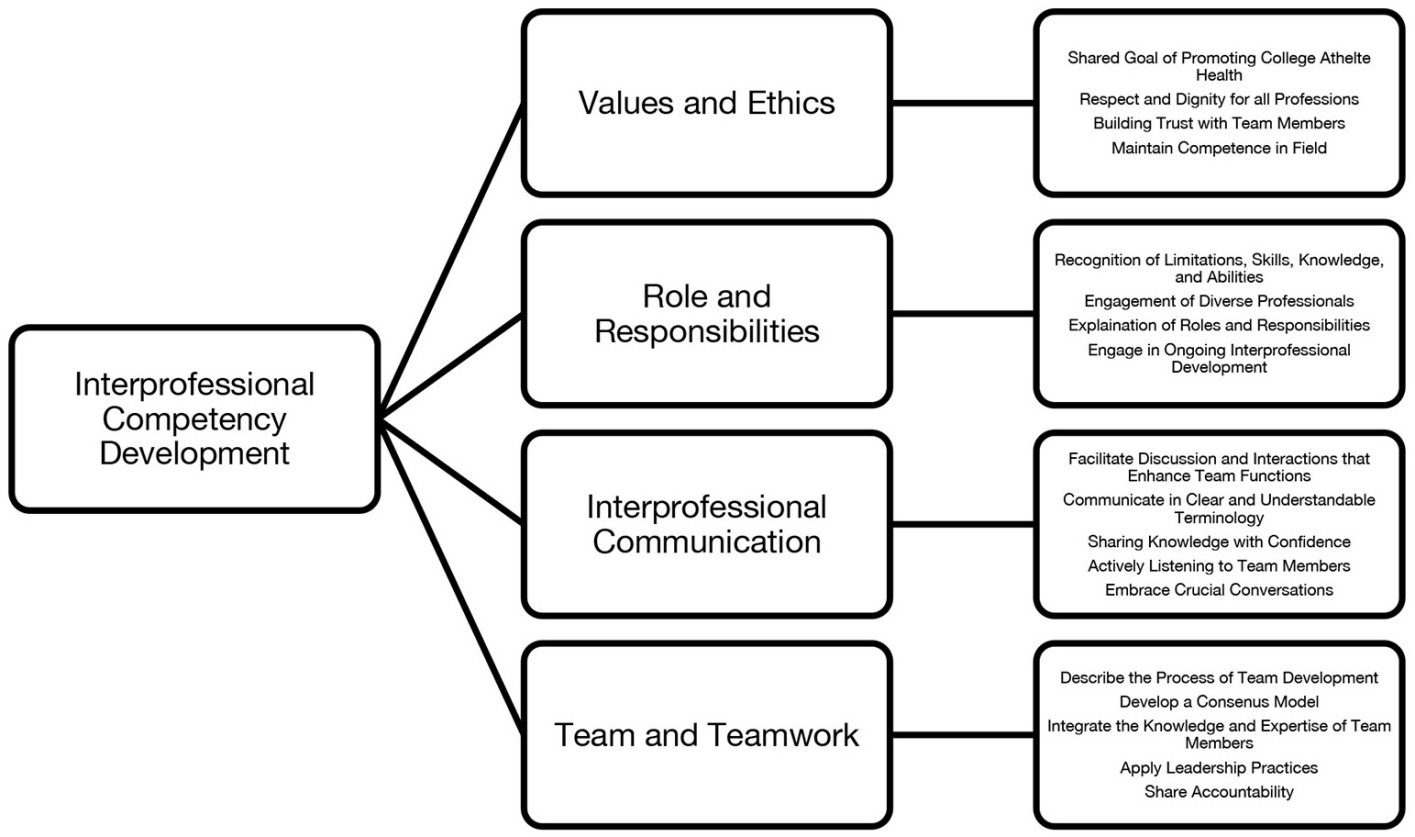
Competency-based development of a college athlete integrated care team.

Effective communication can translate to the development of a team that can accomplish more than if done by a single individual (16). Within a team-based setting, decisions arise from consensus using athlete-focused problem solving. The integration of knowledge from multiple professions informs decisions through a careful examination of multiple lenses and provides a space for constructively managing disagreements and sharing in accountability in the decision-making process. As the team becomes more engaged with one another, evidence emerges and helps to inform effective teamwork and team-based practices see Figure 1 for a model outlining the competency-based development of a college athlete interprofessional care team (16, 20).

## Discussion

At the heart of this competency-based model is the development of a team intentionally created, recognized by others as well as by themselves as having a collective identity and shared responsibility for the mental health of college athletes. One shared responsibility is to improve mental health literacy (13). Jorm et al. (21) define mental health literacy as “knowledge and beliefs about mental disorders, which aid their recognition, management, and prevention” (p. 182). In essence, mental health literacy has three main areas of concentration: (1) knowledge of mental health symptoms and disorders and strategies of self-care, (2) strategies to address public and self-stigma, and (3) create pathways to improve help seeking behaviors (22). A successful interprofessional care team can help mental health literacy become *proactive*, where individuals are not only taught about basic diagnostic information, but also instructed on how to develop social and cognitive skills necessary to address the various determinants of mental health and advocate for change within their sport and larger communities (22).

The access to mental health care remains one of the top priorities for athletic trainers, sport administrators, sport psychiatrists, sport psychologists, sport social workers, and other licensed mental health professionals (13, 23, 24). Members of the interprofessional care team represent diverse professions covering an array of constructs and perspectives that can improve college athlete performance and life outcomes. Some of the constructs and perspectives include: (a) ecological perspectives (athletes are best understood in the context of the systems in which they live), (b) systems theory (exploring the interconnectedness, interdependence, organization, and stability of an elite athlete's relationships), (c) empowerment and strengths perspectives (translating self-efficacy and self-determination to improve both athletic and non-athletic life skills), (d) social learning theory (exploring consequences of thoughts and behaviors on competitive and life success), and (e) models of human development (1). The knowledge base of an interprofessional care team includes theories of biological, psychological, and social development, diversity and cultural competency, interpersonal relationships, group dynamics, mental disorders, addictions, impacts of illness, trauma, or injury, sport performance, psychotropic medication, and the effects of the physical, social, and cultural environment (25, 26). Given the unique challenges facing the college athlete population, the versatility an interprofessional care team is vitally needed.

At the micro level, an interprofessional care team can provide individual services that emphasize and deliver interventions that increase mental health literacy, improve likelihood of earlier intervention and assist college athletes in accessing quality treatment and becoming empowered advocates for their own care [(13, 23, 27), p. 367]. This begins with detailed knowledge of mental health symptoms and disorders, diagnosis and treatment, systemic issues related to sport identity and culture, understanding of sport performance, and developmental factors (22). Furthermore, an interprofessional care team can design services that capture the needs college athletes through various lenses. These efforts allow team members to establish pathways to care, especially clinical care with higher level of expertise theoretically, diagnostically, and through intervention.

At the macro level, an interprofessional care team provides community education and raises awareness of mental health stigma and realities (27). The interprofessional care team can do this through sound pedagogical approaches that emphasize mental health literacy consistently to college athletes and members of their ecological systems (22). These approaches should consider individual and cultural differences associated with particular sports, personal factors, cultural components, environmental determinants, and appropriate formats for capturing the desired target population.

## Conclusion

Our understanding of college athlete mental health continues to evolve. With this evolution will come opportunities to shape the future of care. A key area of future growth is the creation of more interprofessional care models. These models can provide coordinated support, guide professional practice, and acknowledge interprofessional collaboration, outcomes over interprofessional competition. Specifically, interprofessional care models can bridge the values and ethics of various professions, encompass the respect professionals should have for each other and for their clients, and support confidentiality in service delivery. These commonalities will help converge empirical and theoretical knowledge to increase effectiveness of athlete care through interprofessional lenses.

## Author contributions

All authors listed have made a substantial, direct, and intellectual contribution to the work and approved it for publication.
